# Analysis of the Role of Physicians in the Cessation of Cigarette Smoking Based on Medical Specialization

**DOI:** 10.6061/clinics/2018/e347

**Published:** 2018-04-09

**Authors:** Seyhan Dülger, Canan Doğan, Özlem Şengören Dikiş, Eylem Yıldırım, Utku Tapan, İpek Özmen, Birsen Şahin Satılmış, Yavuz Selim İntepe, Birsen Ocaklı, Cevriye Yüksel Kaçan, Ersin Budak, Tekin Yıldız

**Affiliations:** I Pulmonary Diseases Department, Health Sciences University Bursa Yüksek İhtisas Education & Research Hospital, Bursa, Turkey; II Pulmonary Diseases Department, Isparta State Hospital, Isparta, Turkey; III Pulmonary Diseases Department, Bozok University, Yozgat, Turkey; IV Pulmonary Diseases Department, Health Sciences University Suat Seren Chest Disease Education & Research Hospital, İzmir, Turkey; V Pulmonary Diseases Department, Health Sciences University Sureyyapasa Pulmonary Disease Education & Research Hospital, İstanbul, Turkey; VI Pulmonary Diseases Department, Baskent University Zübeyde Hanım Education & Research Hospital, İzmir, Turkey; VII Uludag University Health Sciences Faculty, Bursa, Turkey; VIII Psychology Department, Health Sciences University Bursa Yüksek İhtisas Education & Research Hospital, Bursa, Turkey

**Keywords:** Smoking Cessation, Physician's Role, Dependency

## Abstract

**OBJECTIVE:**

Physicians do not adequately use their unique professional privilege to prevent patients from smoking. The aim of this study was to investigate the type and extent of advice given to patients by physicians of different medical specialties regarding smoking cessation.

**METHODS:**

In total, 317 volunteer physicians were included in this study. The participants rated their attitudes toward the smoking habits of their patients by completing a questionnaire. The approaches used to address the smoking habits of patients significantly differed among physicians working at polyclinics, clinics and emergency service departments (*p*<0.001). Physicians working at clinics exhibited the highest frequency of inquiring about the smoking habits of their patients, while physicians working at emergency service departments exhibited the lowest frequency.

**RESULTS:**

Physicians from different medical specialties significantly differed in their responses. Physicians specializing in lung diseases, thoracic surgery, and cardiology were more committed to preventing their patients from cigarette smoking.

**CONCLUSIONS:**

The role of physicians, particularly pulmonologists and thoracic surgeons, is critical in the fight against cigarette smoking. Promoting physician awareness of this subject is highly important in all other branches of medicine.

## INTRODUCTION

This study was presented as a controversial poster in The Turkish Thoracic Society 20^th^ Annual Congress.

Currently, cigarette smoking is among the highly significant public health problems, and the mortality rates associated with cigarette smoking exceed the total mortality from tuberculosis, human immune deficiency virus and malaria 1. The tobacco epidemic is among the greatest public health threats worldwide, leading to the deaths of 7 million people annually 2. The World Health Organization (WHO) has drawn attention to the potential power of health care workers in the fight against tobacco smoking 3. Even a short-term clinical intervention by a physician has been found to be highly effective in the cessation of cigarette smoking in patients 4-6. However, physicians do not fully exploit this power 7,8. In the U.S.A., more than 70% of cigarette smokers have sought medical consultation for varying reasons 9. These consultations can be useful for short-term clinical interventions by physicians. Physicians who inquire about their patients cigarette smoking habits and advise against smoking can make a significant contribution to public health regardless of their branch of medical specialization and the circumstances of the patients’ visits. The aim of this study was to investigate the approaches used by professionals to the smoking habits of their patients in various medical specializations under various conditions. Thus, the aim of this study was to examine the approaches used to address the smoking habits of patients according to the medical specialty of the physician. Furthermore, to reveal the physicians’ awareness of the harmful effects of cigarette smoking, the physicians were also questioned about diseases associated with their patients’ smoking habits.

## MATERIALS AND METHODS

This study was conducted according to the Helsinki Declaration and was approved by the Bursa Post Graduate Education and Research Hospital Ethical Committee (2011-KAEK-25 2016/14-07).

### Participants

In total, 317 volunteer physicians were included in this study, including 176 (55.5%) males and 141 (44.5%) females working at 6 different health care centers. Participants from polyclinics, specialty clinics and emergency service departments completed a study-specific questionnaire in which they rated their attitudes toward the cigarette smoking habits of their patients and their degree of professional burnout. Preclinical and pediatric physicians in were excluded from the study due to the inapplicability of the questionnaire. Since children are passive smokers, physicians should ask parents about their smoking habits. Childhood illnesses caused or adversely affected by passive smoking differ from adult illnesses. Thus, preparing a different questionnaire for pediatrics and performing a separate study were considered appropriate. Three pulmonologists working at polyclinics specializing in cigarette smoking cessation were also excluded from the study. A preliminary study involving 64 physicians was performed at our hospital, and the results have been published 10. Subsequently, this study was performed to obtain data from different regions and increase participation from other health care centers.

### Questionnaire

To design an appropriate questionnaire, a preliminary version was prepared and assessed by consulting pulmonologists and thoracic surgeons at our hospital. The questionnaire consisted of the following three parts: sociodemographic details of the participating physicians, questions designed to reveal the type and frequency of approaches used by physicians to address the cigarette smoking habits of patients at polyclinics, clinics or emergency service departments (Table 1), and a rating scale (based on the Maslach Burnout Inventory-MBI) to assess professional burnout.

### Statistical Analysis

The relationships among the responses to the questionnaire items, the medical specialization branches, and the types of approach used to address patients’ smoking habits were analyzed using the Statistical Package for Social Sciences (SPSS) (IBM SPSS Statistics for Windows version 23.0, SPSS, Armonk, NY, USA). The numerical data are expressed as the mean±standard deviation (M±SD), and their distribution was assessed by performing a Kolmogorov-Smirnov test. The categorical data are expressed as percentages (%). The mean number of patients visiting physicians with different specialties at polyclinics, clinics and emergency service departments and the median scores of their responses to the study questionnaire were compared using the Mann-Whitney-U test and the Kruskal-Wallis test. Pearson correlation tests were performed to evaluate the relationships among the physicians’ responses to the questionnaire at polyclinics, clinics and emergency service departments, the monthly number of patients treated and the scores on the professional burnout rating scale. Chi-square tests were performed to assess the relationship between the responses to the questionnaire and the demographic information of the physicians. A *p*-value of <0.05 indicated statistical significance.

## RESULTS

The mean age of the participants was 39.61 (±7.65) years. The entire sample consisted of 176 (55.5%) males and 141 (44.5%) females, and 23.4% (n=74) of the participants were smokers. Physicians working at polyclinics who responded that they “specifically” and “always/frequently” inquired about their patients’ cigarette smoking habits constituted 66.88% of the sample, and those who responded that they “frequently or generally” advised their patients to stop smoking constituted 76.58% of the sample.

Statistically significant correlations were not found between the professional burnout ratings and the likelihood of querying about or advising against cigarette smoking among physicians working at polyclinics (*p*=0.48), specialty clinics (*p*=0.37), and emergency service departments (*p*=0.28). In addition, correlations were not observed between the personal cigarette smoking habits of the physicians and their attitudes toward their patients’ smoking habits among physicians working at polyclinics (*p*=0.07), specialty clinics (*p*=0.97) and emergency service departments (*p*=0.45). Unmarried physicians working at emergency services tended to query their patients more frequently regarding their smoking habits than married physicians (*p*=0.026). However, this associated was not statistically significant among physicians working at polyclinics (*p*=0.13) and specialty clinics (*p*=0.19).

**Figure 1:**
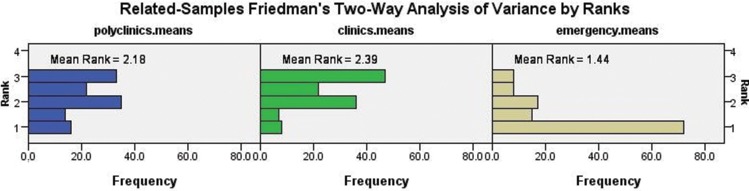
Mean ranks of the responses to the questionnaire at the polyclinics, clinics and emergency service department (*p*<0.001).

**Figure 2:**
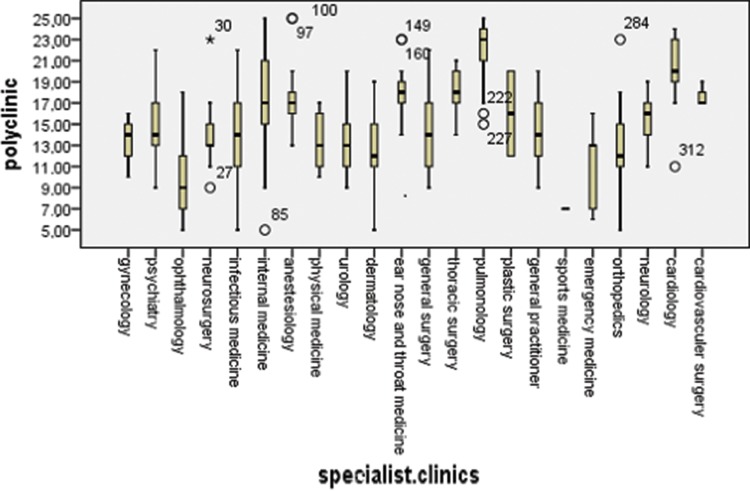
Questionnaire scores of all medical branches at the polyclinics.

**Figure 3:**
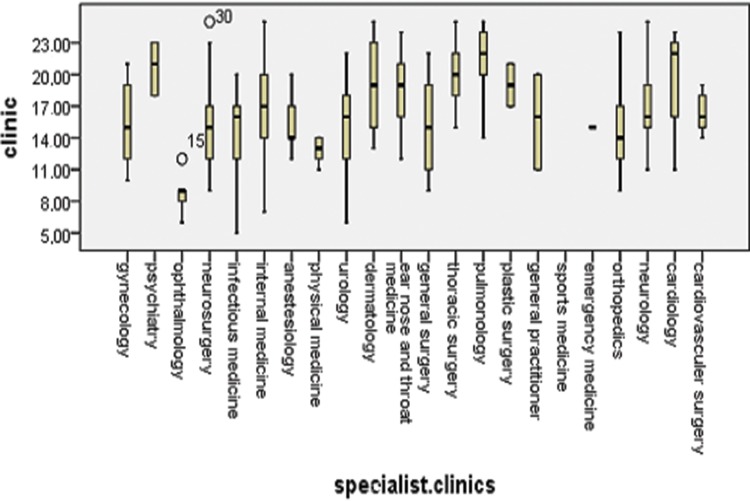
Questionnaire responses of all medical branches at the clinics.

**Figure 4:**
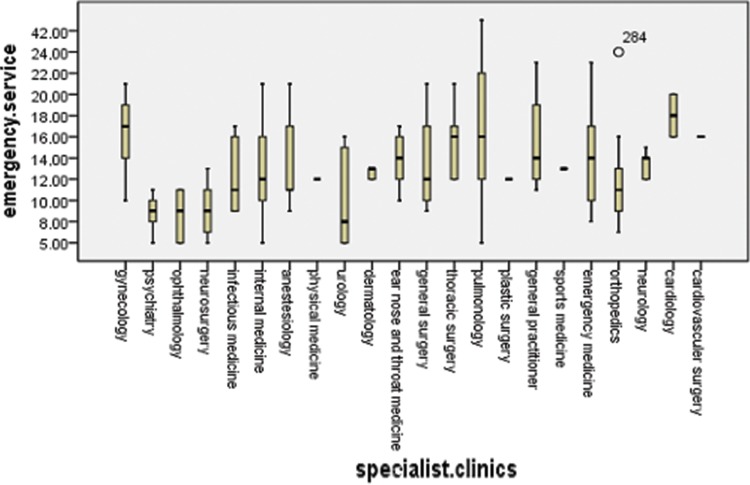
Questionnaire responses of all medical branches at the emergency service department.

Physicians working at polyclinics, clinics and emergency service departments used significantly different approaches to address the cigarette smoking habits of their patients (*p*<0.001). The highest incidence of inquiring about patients’ smoking habits was reported by physicians working at specialty clinics, and the lowest incidence was reported by physicians working at emergency service departments (Figure 1). The variation in the attitudes toward the patients’ smoking habits differed among physicians working at polyclinics (*p*<0.001), clinics (*p*<0.001) and emergency service departments (*p*=0.004). At polyclinics, pulmonologists (4.45±0.53), cardiologists (3.98±0.74), thoracic surgeons (3.57±0.5), ear nose throat (ENT) specialists (3.6±0.51) and cardiovascular surgeons (3.53±0.23) demonstrated a higher sensitivity to their patients’ smoking habits (Figure 2). However, among the specialty clinics, physicians working at eye clinics (1.71±0.38), physical medicine departments (2.53± 0.24) and infectious diseases departments (2.93±0.87) displayed the lower interest in their patients’ smoking habits, and the mean values among the other specialties were similar (Figure 3). At emergency service departments, physicians working in pulmonology (3.58±1.53), cardiology (3.55±0.41), gynecology (3.2±1.11) and thoracic surgery (3.12±0.75) displayed a relatively higher interest in their patients’ smoking habits than physicians working in other disciplines (Figure 4).

At the polyclinics, the high incidences of COPD (67.5%), CAD (55.4%), and CF (50.6%) and the high number of patients in the preoperative stage (63.9%) prompted the physicians to “always” or “frequently” inquire about the smoking habits of their patients. Similarly, at the specialty clinics, the incidence of COPD (44.5%) and CAD (42.3%) and the number of preoperative patients (39.1%) were high, and at the emergency service departments, the incidences of COPD (53.5%) and CAD (44.2%) and the number of patients at the preoperative stage (37.2%) with a smoker’s sociocultural background (37.2%) were high and conditioned the physicians to inquire about the history of the patients’ smoking habits.

## DISCUSSION

Strikingly, we observed a relatively low interest in patients’ smoking habits among physicians working at polyclinics, except for those specializing in pulmonology, thoracic surgery and ENT because these physicians are directly interested in the respiratory system. In particular, ophthalmologists, infectious disease specialists, and general surgeons were not interested in the cigarette smoking habits of their patients. Survey studies investigating this topic are lacking from the literature. King et al. 11 investigated interventions performed by health care providers for smoking cessation, but their study is limited because the medical specializations of the health care providers were not evaluated.

In our study, physicians working at specialty clinics were most likely to inquire about their patients’ smoking habits, followed by physicians working at polyclinics and emergency service departments. Physicians working at specialty clinics spent more time with their patients than physicians working at polyclinics and emergency service departments. In addition, fewer patients visit specialty clinics, which may explain the more favorable results at these clinics. Although similar studies are not available in the literature and despite the relatively short duration of patient contact, we posit that physicians working at emergency service departments should incorporate an assessment of their patients’ smoking habits into the assessment of the presenting complaint of the patient. Even a few minutes spent by the physician on the subject can result in a 5-15% reduction in cigarette smoking 7.

King et al. 11 reported that 87.9% of patients had been asked whether they smoke cigarettes. Demir & Simsek 12 reported that 56.7% of physicians generally or always ask their patients whether they smoke. In the present study, 56% of the physicians working at polyclinics asked about cigarette smoking, indicating that a lack of improvement in our country over the previous 3 years.

King et al. 11 also reported that 65.8% of patients had been recommended to stop smoking by a healthcare professional, while Demir & Simsek 12 and Lindorff & Hill 13 reported that 71.5% and 48.6% of patients, respectively, receive this recommendation. Because it is impossible for the percentage of physicians recommending smoking cessation to exceed the percentage of those who ask about the habit, the result reported by Demir & Simsek 12 may represent 71.5% of physicians who queried, suggesting that 56.7% of all physicians recommend smoking cessation. In our study, 42.36% of the physicians reported that they “always”, “frequently,” or “generally” informed their patients about the harmful effects of cigarette smoking. We were unable to locate this information in the literature.

Approximately 3-5% of the general population quit smoking on their own, and up to 40% of smokers seek consultation at polyclinics for smoking cessation 13. Although the Cigarette Smoking Cessation Polyclinic at our hospital is active 7 days a week, approximately 29.7% of physicians generally” or “frequently” provided referrals. In the study by Demir & Simsek 12, one-third of the participating physicians were unaware of the smoking cessation polyclinic, and only 57.3% of those who were aware of the service (or 38.2% of the total physicians participating in the study) made referrals. Although several studies have suggested that physicians who are not smokers are more interested in their patients’ smoking habits 14–16, we did not observe similar findings. In our study, the physicians who smoked appeared to be more aware of smoking as an issue despite being smokers.

In this study, no significant differences were observed in the responses to the questionnaires based on the physicians’ gender; however, compared to married physicians, unmarried physicians working at emergency service departments were more aware of their patients’ smoking habits. The relatively lower number of physicians employed at emergency service departments may have contributed to this finding. Furthermore, this positive finding may be due to the younger age and more recent graduation of the unmarried physicians.

Patients with COPD, CAD, or CF and patients presenting for preoperative consultation appeared to remind the physicians to inquire about smoking habits. At emergency service departments, the sociocultural background of the patients also prompted the physicians to obtain the patients’ smoking history. The relatively low rates of inquiry about cigarette smoking habits in patients with diabetes mellitus and collagen tissue diseases, which are adversely affected by smoking, are alarming. We did not identify any reports related to this subject in the literature. However, we believe that physicians should be specifically informed of the relationship between cigarette smoking and chronic illnesses as a part of their education.

One limitation of this study is the self-reported nature of the data, which were based on a study-specific questionnaire, and direct observations of the participants at the polyclinics, clinics and emergency service departments were not performed. Furthermore, feedback from the patients regarding the approaches used by the physicians to address their smoking habits was not collected; only physicians were questioned about the approach used to address their patients’ smoking habits. Pediatricians caring for children who are passive smokers were also excluded from this study.

Cigarette smoking is a serious threat to public health in this century, and physicians must fully perform their duty in fighting against this problem. This study revealed that physicians of various medical specialties are not equally aware of and informed about the appropriate approaches that can be used to address patients who are smokers. Physicians are unlikely to ask about smoking in patients who do not have COPD, CAD and CF. Furthermore, physicians do not have basic knowledge regarding the effects of cigarettes on the course of many illnesses and the efficacy of many drugs. We believe that the education of physicians of all medical specialties should emphasize the issue of cigarette use and dependence.

## AUTHOR CONTRIBUTIONS

Seyhan Dülger S generated the hypothesis, planned the methodology, performed the statistical analysis, and wrote the manuscript. Doğan C, Yıldırım E, Tapan U, Özmen İ, Satılmış BŞ, İntepe YS and Ocaklı B performed the data collection at their center, data compilation, and literature review. Dikiş ÖŞ performed data collection at her center, data compilation, literature review, and critical review of the manuscript. Kaçan CY and Budak E performed the survey preparation, data collection, data compilation, and literature review. Yıldız T planned the methodology, and organized and critically reviewed the manuscript.

## Figures and Tables

**Table 1:** Questionnaires distributed to physicians working at polyclinics, clinics and emergency service departments.

A- Are you following up an inpatient at any clinic? (If your answer is ‘’Yes’’, answer the questions on this page. If your answer is ‘’No’’, you may continue with the following pages)
1. What is the mean number of patients you have seen at the polyclinics in a single month?
2. I query whether the polyclinic patients smoke or do not smoke cigarettes.
A) Never B) Rarely C) Generally D) Frequently E) Always
3. I inform the polyclinic patients on the harmful effects of cigarette smoking.
A) Never B) Rarely C) Generally D) Frequently E) Always
4. I Advise the polyclinic patients to give up smoking cigarettes.
A) Never B) Rarely C) Generally D) Frequently E) Always
5. I refer the polyclinic patients to the Cigarette Smoking Cesation polyclinic after the completion of their treatment.
A) Never B) Rarely C) Generally D) Frequently E) Always
